# Mediation of Potato–Potato Cyst Nematode, *G. rostochiensis* Interaction by Specific Root Exudate Compounds

**DOI:** 10.3389/fpls.2020.00649

**Published:** 2020-06-10

**Authors:** Juliet Ochola, Laura Cortada, Margaret Ng’ang’a, Ahmed Hassanali, Danny Coyne, Baldwyn Torto

**Affiliations:** ^1^Behavioural and Chemical Ecology Unit, International Centre of Insect Physiology and Ecology, Nairobi, Kenya; ^2^Department of Chemistry, Kenyatta University, Nairobi, Kenya; ^3^Nematology Research Unit, Department of Biology, Ghent University, Ghent, Belgium; ^4^International Institute of Tropical Agriculture, Nairobi, Kenya

**Keywords:** *Globodera* spp., hatching factors, potato root exudates, semiochemicals, steroidal glycoalkaloids

## Abstract

Potato (*Solanum tuberosum*) is a widely consumed staple food crop worldwide whose production is threatened by potato cyst nematodes (PCN). To infect a host, PCN eggs first need to be stimulated to hatch by chemical components in the host root exudates, yet it remains unknown how most root exudate components influence PCN behavior. Here, we evaluated the influence of eight compounds identified by LC-QqQ-MS in the root exudate of potato on the hatching response of the PCN, *Globodera rostochiensis* at varying doses. The eight compounds included the amino acids tyrosine, tryptophan and phenylalanine; phytohormones zeatin and methyl dihydrojasmonate; steroidal glycoalkaloids α-solanine and α-chaconine and the steroidal alkaloid solanidine. We additionally tested two other Solanaceae steroidal alkaloids, solasodine and tomatidine, previously identified in the root exudates of tomato, an alternative host for PCN. In dose-response assays with the individual compounds, the known PCN hatching factors α-chaconine and α-solanine stimulated the highest number of eggs to hatch, ∼47 and ∼42%, respectively, whereas the steroidal alkaloids (aglycones), solanidine and solasodine and potato root exudate (PRE) were intermediate, 28% each and 21%, respectively, with tomatidine eliciting the lowest hatching response 13%. However, ∼60% of the hatched juveniles failed to emerge from the cyst, which was compound- and concentration-dependent. The amino acids, phytohormones and the negative control (1% DMSO in water), however, were generally non-stimulatory. The use of steroidal glycoalkaloids and their aglycones in the suicidal hatching of PCN offers promise as an environmentally sustainable approach to manage this pest.

## Introduction

Potato cyst nematodes (PCN) have been of great economic importance to potato production since their introduction to Europe in the mid 1880’s. They originate from the Andean region in South America (Grenier et al., 2010) but have subsequently spread to other potato-growing regions of the world (Hockland et al., 2012). The PCN species *Globodera rostochiensis* and *Globodera pallida* are among the most regulated quarantine nematode pests globally, with a potential to cause potato (*Solanum tuberosum*) yield losses of up to 70% (Turner and Subbotin, 2013). In the United Kingdom, PCN alone causes up to 9% potato production losses, corresponding to a market value of around €43 million annually (Sasaki-Crawley, 2013). In the rest of Europe, the market value of losses is approximately seven times higher (€300 million annually) (Moxnes and Hausken, 2007).

Recently, both species of PCN have been detected in Kenya (Mwangi et al., 2015; Mburu et al., 2018), where potato is the second most important food crop (CIP, 2019). Potato, a crop which is grown mainly by smallholder farmers, provides more calories per cultivated area than any other crop and a source of income generation to millions of people (VIB, 2019). However, in recent years, potato production has experienced a 61% decline, even though the potato cultivation area has increased (Food and Agriculture Organization [FAO], 2017). Several factors including the lack of quality seed, poor agronomic practices and PCN and diseases are major contributors to this decline (Kaguongo et al., 2014). Recently, the potential impact of *G. rostochiensis* has been further highlighted, which has now been confirmed to be widespread across Kenya (Mwangi et al., 2015; Mburu et al., 2020) and present within regions in Rwanda (Niragire et al., 2019) and Uganda (Cortada et al., 2020). As such, PCN poses a major threat to potato production on a regional level (Mburu et al., 2018).

Infection by PCN diverts the host plant nutrients to the nematode and causes physical damage to plant tissue as the nematodes migrate through the roots, leading to potential secondary infections (Jones et al., 2013). This effect translates to reduced water and nutrient uptake, resulting in decreased crop yield. To ensure effective penetration of root tissue, the nematodes secrete effectors, comprised of proteins, which can alter the host cell structure. Additionally, they suppress host plant defenses (Haegeman et al., 2012; Yang et al., 2019), increasing the susceptibility of the plant to attack by other pests and pathogens.

Current PCN management strategies include the use of botanicals, synthetic chemical nematicides, biological control agents, genetic resistance, crop rotation, trap crops, fallow rotation, organic soil amendments and/or intercropping. These management strategies have been implemented with varying degrees of success, while PCN remain a persistent and economic threat to the potato industry. Most synthetic chemical nematicides that were previously effective have been withdrawn from use due to their adverse environmental and toxic effects (Khalil, 2013). Despite resistant cultivars having been identified as the most effective method for cyst nematode management, the species-specific resistance response of some of the resistance genes (*R*-genes) to certain nematode species allows other PPN species, when present, to reproduce. Cyst nematodes tend to have a limited host range and require specific chemical cues from host plants to stimulate hatch and for host location. A characteristic feature of cyst nematodes is their ability to endure periods of adverse conditions or the absence of hosts, as a cyst. This diapausing structure limits the effectiveness of crop rotation or fallowing, or even chemical management as the nematodes are inactive and are protected by the cyst structure, making them particularly pervasive pests.

Given the importance of potatoes in East Africa, there is an urgent need to establish PCN management options that are more effective and environmentally sustainable for small holder farmers. Innovative solutions, such as exploiting semiochemical based tools to disrupt the nematode life cycle could prove effective. One promising avenue appears to be the induction of ‘suicidal hatch’ using naturally occurring phytochemicals (Devine and Jones, 2000, 2001; Devine et al., 2001). The hatching of the infective juveniles (J2s) from encysted eggs is triggered by chemical signals originating from the host plant roots. The hatch is initiated by alteration of the eggshell membrane, which is a calcium-mediated process that involves either hatching factors from the host root exudates binding or displacement of internal calcium ions. Although, some studies have identified compounds in host root exudates, such as the triterpenoid solanoeclepin A that stimulate PCN hatch (Mulder et al., 1996), their practical application for PCN management is yet to be realized. The structural complexity of solanoeclepin A makes it expensive and challenging to synthesize in sufficient amounts for use in the field (Tanino et al., 2011).

Other PCN hatching factors identified from potato root exudates include the steroidal glycoalkaloids (SGA) α-solanine and α-chaconine (Devine et al., 1996). Generally, potato and other Solanaceae plants are known to produce steroidal glycoalkaloids, their aglycones (steroidal alkaloids) and other classes of compounds (Shakya and Navarre, 2008; Distl and Wink, 2009). However, most of these compounds are yet to be screened for PCN hatching response. In the current study, we profile the chemistry of the root exudate of a PCN-susceptible and widely grown potato cultivar, ‘Shangi,’ in Kenya (Mburu et al., 2020), and screen specific identified compounds for induction of PCN suicide hatch.

## Materials and Methods

### Plants

Seed potatoes of cv. ‘Shangi’ were planted in 2 L plastic pots (13 cm base diameter, 17 cm top diameter and 15 cm deep) perforated at the base and filled with sterilized sand, autoclaved for 40 min at 121°C (Astell Scientific autoclave, United Kingdom). The plants were grown in a screenhouse at the International Centre of Insect Physiology and Ecology (*icipe*) Duduville Campus, Nairobi, Kenya (1.2219° S, 36.8967° E) at 23 ± 3°C and relative humidity 60–70%. The plants were watered twice a week using a nutrient solution prepared according to Lambert et al. (1992) for 5 weeks before use in the study.

### Potato Root Exudates

Potato root exudates of 5 weeks old plants, were collected using the dipping method (Kirwa et al., 2018). Briefly, the plants were gently removed from the soil, the roots washed in tap water to remove sand particles and then rinsed in distilled water. Batches of five plants were combined to constitute one replicate and the roots immersed in 500 ml of distilled water in a 2 L glass beaker to collect the potato root exudates. This was done in three replicates. The beaker was wrapped with aluminum foil to prevent photodegradation and the setup left for 24 h. The collected exudates were filtered and freeze-dried (SP Scientific, VirTis Advantage) to dryness and weighed. The potato root exudate was kept at −80°C until used for chemical analysis and hatching bioassays.

### Nematodes

Potato cyst nematodes *G. rostochiensis* samples were obtained from soil from a freshly harvested farm in Nyandarua County, Kenya (00.78537° S, 036.60429° E). The soil was air-dried and cysts extracted by floatation using a Fenwick can (EPPO, 2013). The cysts were collected on a milk filter paper, dried, hand-picked under a stereomicroscope (LEICA M125, Singapore) and sorted based on color. Freshly formed cysts were used for the hatching experiments, which, under Kenyan conditions appear to be hatching without the need for diapause and which is currently being investigated further.

### Chemicals

LC-MS grade methanol (LC-MS LiChrosolv^®^, Merck (≥99.97%), formic acid (98–100%), water (LC-MS Chromasolv), *trans*-zeatin (≥97%), solasodine (≥95%), tomatidine hydrochloride (≥85%), α-solanine (≥95%), solanidine (≥95%), phenylalanine (≥98%), tyrosine (≥98%), tryptophan (≥95%), methyl dihydrojasmonate (≥96%), and DMSO (≥99.9%) were purchased from Sigma-Aldrich (St. Louis, MO, United States) and α-chaconine (≥95%) (PhytoLab, Germany).

### Chemical Analysis of the Potato Root Exudates

To determine the chemical composition of potato root exudates, 10 mg of the freeze-dried root exudates was dissolved in 1 ml of 30% methanol in ddH_2_O. The sample was then vortexed for 10 s, sonicated for 30 min, and centrifuged at 14,000 rpm for 10 min. The supernatant was diluted to 1 mg/ml and transferred into a sample vial and 0.1 μl analyzed using an Ultra Performance Liquid Chromatography coupled to a triple quadrupole tandem mass spectrometry (UPLC-QqQ-MS/MS). Chromatographic separation was performed on a ACQUITY UPLC I-class system (Waters Corp., Milford, 151 MA) fitted with an ACQUITY UPLC BEH C18 column (2.1 mm × 150 mm, 1.7 μm particle size; Waters Corp., Wexford, Ireland), that was heated to 45°C. The autosampler tray was cooled to 5°C. The mobile phase comprised of water acidified with 0.01% formic acid (solvent A) and methanol (solvent B) and followed a gradient system. The gradient system used was 0–2 min, 5% B, 2–4 min, 40% B, 4–7 min, 40% B, 7–8.5 min 60% B, 8.5–10 min 60% B, 10–15 min, 80% B, 15–19 80% B, 19–20.5 min, 100% B, 20.5–23 min, 100% B, 23–24 min 95% B, 24–26 min, 95% B. The flow rate was held constant at 0.2 ml/min.

The UPLC was interfaced with an electrospray ionization (ESI) Waters Xevo TQ-S operated in full scan MS in both positive and negative ionization modes. Data were acquired over the m/z range 100–2,000 with a capillary voltage of 0.5 kV, sampling cone voltage of 30 V, source temperature 150°C and desolvation temperature of 120°C. The nitrogen desolvation flow rate was 600 L/h.

Data was acquired using MassLynx version 4.1 SCN 712 (Waters). Potential assignments of compounds were determined after the generation of the mass spectrum for each peak, establishing the molecular ion peaks using adducts, common fragments, literature and where available, confirmed with authentic samples through co-injections. All the samples were analyzed in triplicate, with each replicate collected from different batches of plants.

### Hatching Assays

We tested 10 synthetic standards: the steroidal alkaloids solanidine, solasodine, and tomatidine hydrochloride; steroidal glycoalkaloids α-solanine and α-chaconine; amino acids, tryptophan, tyrosine and phenylalanine; and phytohormones, zeatin and methyl dihydrojasmonate. PCN *in vitro* hatching bioassays were conducted according to the protocol used by Twomey et al. (1995). The standards were diluted to five concentrations: 0.2, 0.4, 0.6, 0.8, and 1 μg/ml from a stock of 1 mg/ml in 10% DMSO. A 200 μl aliquot of each test solution was added to each well of a Linbro^®^ 96 multi-well sterile plate containing five cysts per well, pre-soaked in water for 5 days (Twomey et al., 1995). Each concentration was tested in six replicates. Potato root exudate and 1% DMSO in water were used as the positive and negative control, respectively.

The 96-well plate was covered and placed in the dark at 20 ± 3°C. The emerging J2 were counted weekly and the test solutions replenished following each count. After 5 weeks, the remaining eggs within each cyst were assessed for viability using Nile blue A stain [(5-aminobenzo[a]phenoxazin-9-ylidene)-diethylazanium;sulfate - Sigma-Aldrich, India], which stains dead/non-viable eggs but not live ones (Ogiga and Estey, 1974).

Hatching activity for each experimental treatment was expressed as cumulative percentage (%) of hatched J2 recovered from the wells and the encysted hatched J2 recovered from the cysts. The total number of viable eggs was the sum of hatched J2 plus the viable eggs recovered from the cyst.

### Data Analysis

A hatching index (HI) for all tested compounds was calculated according to the formula:

HI=(TH-CH)/(TH+CH)×100

where, TH is the number of J2 hatching stimulated by a test compound and CH is the number of J2 stimulated by the positive control, potato root exudate. A positive value in the hatching index indicates that a higher proportion of the J2 hatched on stimulation by the compounds, compared to the control, whereas a negative value indicates the opposite effect. Plots of hatching index were made separately for the J2 that emerged from the cyst and those that remained encysted. The data on the hatched J2 were analyzed using a generalized linear model with a binomial distribution. Using the control potato root exudate as a reference category, the odds ratios (ORs), a measure of the likelihood that PCN responded to the other treatments instead of the control were estimated including Confidence Interval (CI) and corresponding *p*-values. With OR for the control set at 1, values above this indicates better hatching response and values below underperformance of the treatments, relative to the positive control. Differences between the compounds were evaluated using a test of proportions. Significant differences between the emerged/encysted J2s hatching indices and the positive control was determined using one-sample *t*-test and one-sample wilcoxon signed rank test depending on the normality of the data. All the analyses were conducted on R version 3.5.2 using a 5% significance level.

## Results

### Chemical Analysis of Potato Root Exudates

**FIGURE 1:**
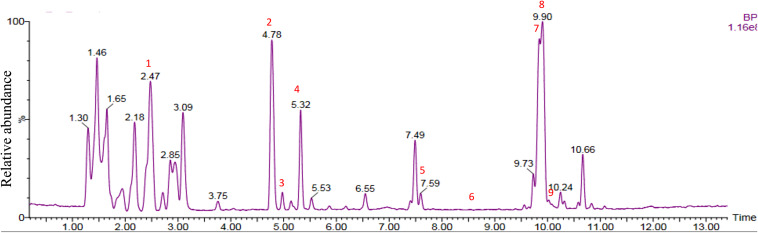
LC-QqQ-MS chromatogram of potato root exudates from 5-week-old plants with identified compounds.

Chemical analysis of the potato root exudates, by LC-QqQ-MS, identified 9 compounds belonging to four classes; amino acids, phytohormones, triterpenoid and steroidal alkaloids (Figure 1 and Table 1). Additionally, the chemical profile of the root exudate showed several unidentified components (Figure 1).

**TABLE 1:** LC-QqQ-MS fragments of identified compounds.

Peak No.	*t*_R_ (min)	Compound	Structures	Class of compound	(M + H)	(M − H)	Positive mode Fragmentation	Reference
1	2.41	Tyrosine^b^	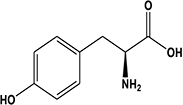	Amino acid	182.2	180.1	119.0, 136.0, 147.0, 165.1	El Aribi et al., 2004; Zhang et al., 2019
2	4.76	Phenylalanine^b^	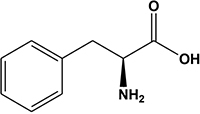	Amino acid	166.1	164.0	103.0, 120.0, 149.1, 131.0	Piraud et al., 2003
3	5.18	Zeatin^b^	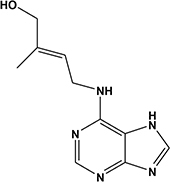	Phytohormone	220.3	218.2	202.2, 137.0	Kirwa et al., 2018
4	5.31	Tryptophan^b^	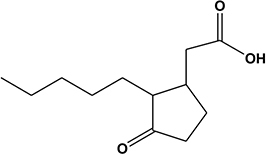	Amino acid	205.2	203.1	188.2, 146.1, 159.2	El Aribi et al., 2004; Zhang et al., 2019
5	7.57	Methyl-dihydrojasmonate^b^	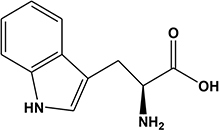	Phytohormone	227.3	225.1	209.2, 167.1	Zeng et al., 2018
6	8.67	Solanoeclepin A^b^	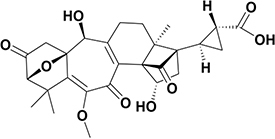	Tetranortriterpenoid	499.6	497.1	–	Sasaki-Crawley, 2013
7	9.92	α-Chaconine^a^	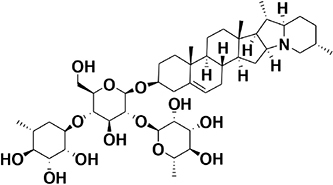	Steroidal glycoalkaloid	852.6	850.9	706.7, 560.7, 398.6	Stobiecki et al., 2003; Cataldi et al., 2005
8	9.90	α-Solanine^a^	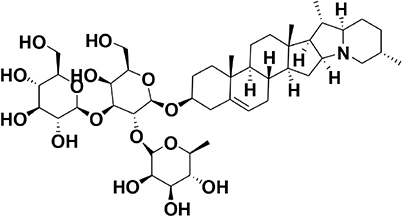	Steroidal glycoalkaloid	868.8	866.9	722.9, 706.7, 560.7, 398.6	Stobiecki et al., 2003; Cataldi et al., 2005
9	10.01	Solanidine^a^	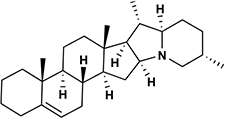	Steroidal alkaloid	398.6		–	Shakya and Navarre, 2008

*^*a*^Identified by co-injection with authentic samples. ^*b*^Identified by comparison to literature.*

The amino acids identified included tyrosine (1), phenylalanine (2) and tryptophan (3). Phytohormones, zeatin (4) and methyl dihydrojasmonate (5) and a tetranortriterpenoid, solanoeclepin A (6) was also identified. The steroidal glycoalkaloids (SGA) α-chaconine (7) and α-solanine (8) with their aglycone, solanidine (9) were also detected in the potato root exudate. All the compounds were identified in both positive and negative ionization modes with key fragments illustrated in Table 1.

### Hatching Response of PCN to the Synthetic Compounds

**FIGURE 2:**
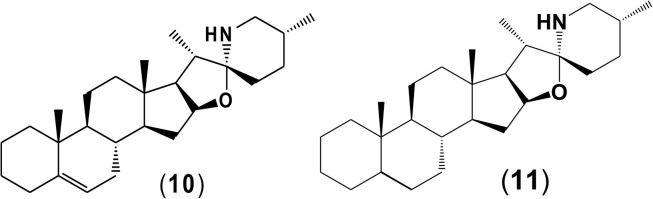
Structures of steroidal alkaloids solasodine (10) and tomatidine (11).

**FIGURE 3:**
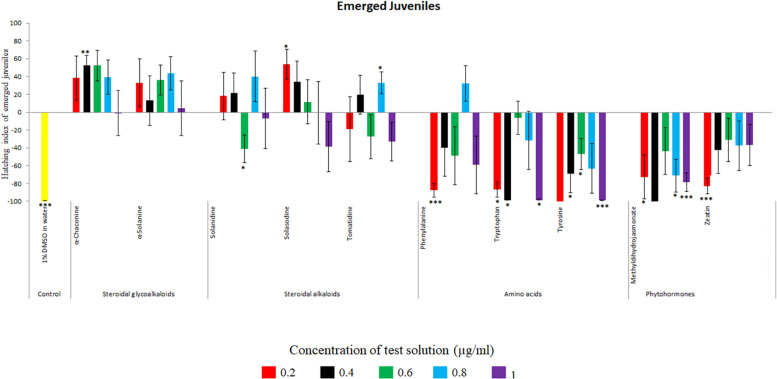
Hatching index of emerged J2s of PCN, *G. rostochiensis* in response to ten compounds compared to the positive control, potato root exudates at five concentrations (μg/ml). Error bars indicate standard error of the mean (SEM). Treatments with an asterisk above the bars indicate a significant difference from the positive control. The level of significance is indicated by ^∗^ for *p* < 0.05, ^∗∗^*p* < 0.01, ^∗∗∗^*p* < 0.001.

**FIGURE 4:**
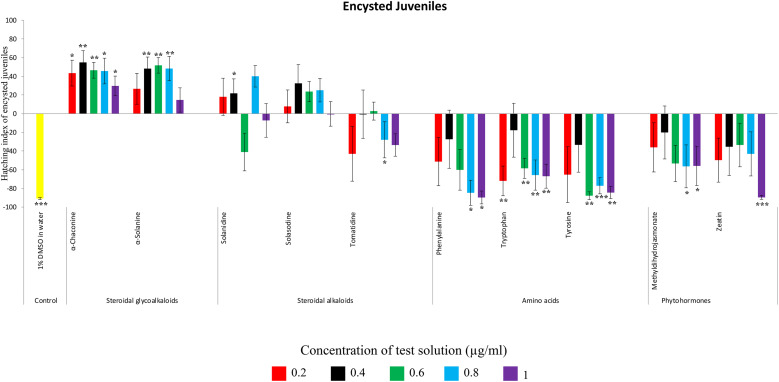
Hatching index of encysted hatched J2s of PCN, *G. rostochiensis* in response to ten compounds compared to the positive control, potato root exudates at five concentrations (μg/ml). Error bars indicate standard error of the mean (SEM). Treatments with an asterisk above the bars indicate a significant difference from the positive control. The level of significance is indicated by ^∗^ for *p* < 0.05, ^∗∗^*p* < 0.01, ^∗∗∗^*p* < 0.001.

Potato cyst nematode *G. rostochiensis* hatching response over the range of concentrations tested was highest in SGAs α-chaconine (7) and α-solanine (8), ∼47 and ∼42%, respectively, intermediate for the steroidal alkaloids (aglycones), solanidine (9), solasodine [Figure 2 (10)] and tomatidine [Figure 2 (11)] and PRE, ∼28, ∼28, ∼13, and 21% respectively, and lowest for the phytohormones zeatin (4) and methyl dihydrojasmonate (5), amino acids tyrosine (1), phenylalanine (2) and tryptophan (3) and the negative control (1% DMSO in water), <10%. For the SGAs, PCN was more sensitive to lower concentrations of α-chaconine (7) at 0.2 μg/ml (χ^2^ = 10.39, df = 1, *p* < 0.05) and at 0.4 μg/ml (χ^2^ = 90.12, df = 1, *p* < 0.001) than α-solanine (8) (Figures 3, 4 and Table 2).

**TABLE 2:** Comparisons of PCN hatching response to 10 compounds relative to potato root exudate (positive control).

Treatment		Concentration (μg/ml)				
		0.2 μg/ml	0.4 μg/ml	0.6 μg/ml	0.8 μg/ml	1 μg/ml
α-Solanine	*p*-value	0.108	0.046	0.000	0.001	0.390
	OR (95% CI)	2.14 (0.86–5.61)	2.78 (1.05–8.00)	5.25 (2.62–11.19)	5.15 (2.02–14.29)	1.38 (0.67–2.88)
α-Chaconine	*p*-value	0.002	0.000	0.000	0.013	0.461
	OR (95% CI)	4.19 (1.69–10.90)	5.75 (2.25–16.30)	5.28 (2.59–11.20)	3.44 (1.34–9.57)	1.32 (0.64–2.74)
Solanidine	*p*-value	0.466	0.035	0.497	0.092	0.389
	OR (95% CI)	1.42 (0.55–3.79)	2.93 (1.11–8.44)	1.32 (0.60–2.95)	2.34 (0.89–6.58)	0.74 (0.34–1.60)
Solasodine	*p*-value	0.485	0.028	0.084	0.159	0.444
	OR (95% CI)	1.41 (0.54–3.76)	3.07 (1.17–8.81)	1.95 (0.93–4.25)	2.06 (0.77–5.83)	0.51 (0.22–1.13)
Tomatidine	*p*-value	0.032	0.954	0.808	0.705	0.102
	OR (95% CI)	0.20 (0.03–0.76)	1.03 (0.33–3.26)	0.91 (0.38–2.11)	0.79 (0.25–2.51)	0.36 (0.15–0.84)
Phenylalanine	*p*-value	0.000	0.767	0.010	0.008	0.021
	OR (95% CI)	0.12 (0.03–0.34)	0.79 (0.14–3.86)	0.29 (0.10–0.70)	0.27 (0.09–0.66)	0.09 (0.02–0.27)
Tryptophan	*p*-value	0.000	0.192	0.100	0.003	0.000
	OR (95% CI)	0.06 (0.00–0.23)	0.23 (0.01–1.64)	0.51 (0.22–1.11)	0.23 (0.08–0.57)	0.06 (0.01–0.18)
Tyrosine	*p*-value	0.001	0.195	0.004	0.010	0.000
	OR (95% CI)	0.06 (0.00–0.23)	0.23 (0.01–1.64)	0.13 (0.02–0.43)	0.24 (0.07–0.65)	0.03 (0.00–0.12)
Methyl dihydrojasmonate	*p*-value	0.035	0.006	0.074	0.039	0.000
	OR (95% CI)	0.31 (0.10–0.88)	0.25 (0.09–0.64)	0.42 (0.15–1.05)	0.15 (0.01–0.71)	0.09 (0.02–0.25)
Zeatin	*p*-value	0.007	0.010	0.209	0.619	0.000
	OR (95% CI)	0.15 (0.03–0.52)	0.28 (0.12–0.72)	0.57 (0.23–1.34)	0.76 (0.24–2.27)	0.15 (0.02–0.26)

*Values are the p-values, odds ratios (OR) and confidence intervals (CI) from a comparison of mean percentage hatch of 6 replicates from the test solutions and potato root exudate at different concentrations.*

Potato cyst nematodes hatching index for the aglycones varied with the concentration of the compound. Solasodine (10), elicited a higher emerged J2 response across the different concentrations than solanidine (9): at 0.2 μg/ml; (χ^2^ = 100.25, df = 1, *p* < 0.001), at 0.4 μg/ml; (χ^2^ = 32.70, df = 1, *p* < 0.001), with tomatidine (11) at 0.2 μg/ml; (χ^2^ = 37.92, df = 1, *p* < 0.001); and 0.6 μg/ml; (χ^2^ = 4.96, df = 1, *p* < 0.05) eliciting the lowest response (Figure 3). However, PCN response to the aglycones was dominated by encysted than emerged J2, with comparable responses to solasodine and solanidine (Figure 4 and Table 2).

Of the amino acids, phenylalanine (2) stimulated a higher J2 hatch compared to tyrosine (1) and tryptophan (3) but only for the emerged J2 (Figure 3 and Table 2), whereas there was no detectable stimulation of encysted J2 hatch for all the amino acids. A similar pattern was observed for the phytohormones and the negative control (1% DMSO in water) (Figures 3, 4 and Table 2).

## Discussion

Root exudate is an important factor that influences interactions with organisms in the rhizosphere (Koo et al., 2005; Canarini et al., 2019). It consists of organic compounds that include both primary (e.g., sugars, amino acids, and organic acids) and secondary metabolites (e.g., alkaloids, flavonoids, terpenes, and phenolics) that are passively released into the rhizosphere through the roots of living plants across a concentration gradient (Grayston et al., 1997; Vranová et al., 2013). Root exudates are utilized by soil dwelling microbes for various biological processes and they can also function as semiochemicals (chemical attractants, repellants, hatching factors, and hatching inhibitors), growth inhibitors and growth promoters, among other functions (Walker et al., 2003). Our chemical analysis of potato root exudate identified different classes of compounds, including amino acids, phytohormones, steroidal glycoalkaloids and steroidal alkaloids reflecting the findings of previous studies (Koo et al., 2005; Li et al., 2013; Kirwa et al., 2018), and several unidentified components, whose identities and biological roles would require further research.

In the present study, α-solanine (7) and α-chaconine (8) elicited the highest PCN hatching response, consistent with the previous findings (Devine et al., 1996; Byrne et al., 1998; Devine and Jones, 2000). Structurally, α-solanine and α-chaconine are chemically similar. They bear the same aglycone, solanidine, which is attached to three different sugar moieties in the two different SGAs. For instance, the triose of α-solanine consists of two six-carbon sugars, D-glucose and D-galactose and one five-carbon sugar L-rhamnose. On the other hand, the triose of α-chaconine consists of one six-carbon sugar D-glucose and two five-carbon sugar L-rhamnose (Ghisalberti, 2006). The fact that the two SGAs elicited a similar hatching response pattern, which varied with concentration suggest that the sugar type linked to the aglycone may influence J2 stimulation response. Although the two SGAs are highly oxygenated and polar, the sugar types and their configuration may determine their binding to receptors via hydrogen bonding to stimulate J2 hatch (Torto et al., 2018a). It appears that α-chaconine may have the correct configuration to bind to the receptors to stimulate J2 hatch at lower concentrations, whereas higher concentrations of α-solanine may be needed to saturate and bind to the hatching receptors. Since a sialoglycoprotein binding site for hatching in the eggshell membrane of *G. rostochiensis* has been identified (Atkinson and Taylor, 1983), it would be interesting to identify SGA binding sites to determine the optimal concentrations needed for PCN to discriminate between different SGAs. However, it is interesting that the majority of J2 that hatched in response to the SGAs remained encysted. Several reasons may account for this observation. Firstly, this suggests that additional chemical cues are needed to stimulate optimal emergence (e.g., hatching and post-hatch movement) and to complete the host location process of J2s. Secondly, the SGAs could be binding to the emerged J2 receptors, thereby activating a feedback signal that blocks additional emergence of the encysted J2s. Thirdly, the SGAs could also stimulate a sudden burst in the hatching of J2s, leading to overcrowding and movement through the vulval fenestra of the cyst. Future work should investigate all these possible scenarios. Previously, SGAs have been associated with growth inhibition of various bacterial (Seipke and Loria, 2008) and fungal species (Munafo and Gianfagna, 2011). The fact that we found SGAs in the potato root exudate of plants grown in sterilized autoclaved sand, suggests that the plant may be priming itself against microbial attack. As such, it appears that the PCN has, over time, evolved to ‘eavesdrop’ into the plant defense priming system to hatch in response to varying concentrations of SGAs, as demonstrated in the present study.

Although PCN hatched in response to the steroidal alkaloids (aglycones), it was lower than for the SGAs. This is likely because they are less oxygenated and therefore less polar than the SGAs. As with the SGAs, most of the hatched J2 remained in the cyst, confirming that additional cues are required to stimulate optimal emergence. However, of the aglycones, tomatidine (11) elicited the lowest hatching response. Structurally, tomatidine is less polar than solasodine and solanidine because the latter two bear an olefinic bond in ring B of the molecule. Consequently, future studies should elucidate structure-activity relationships, such as the influence of oxygenation and presence of olefinic bonds in triterpenoids identified in the potato root exudate and the exudates of other host plants in PCN hatching response. A recent study identified solasodine and tomatidine in the root exudate of tomato, an alternative host for PCN, which induced significant stylet thrusting in the plant parasitic nematode, *Meloidogyne incognita*, even though they did not elicit attraction (Kirwa et al., 2018). In another study, pre-exposure of *M. incognita* to the quinolone alkaloids waltherione A and waltherione E, isolated from the aerial part of *Triumfetta grandidens*, significantly inhibited egg hatch (Jang et al., 2015). These findings demonstrate the parsimonious role of specific host root exudate compounds on the behavior of different plant parasitic nematodes- specific SGAs and their aglycones as hatching factors in *G. rostochiensis*, but also serve as egg hatching inhibitors and J2 stylet thrusting stimulants in *M. incognita*. Hence, it would be interesting to further examine these compounds at the same concentrations, either individually or in a blend, to elucidate their full role in the behavior of different species of plant parasitic nematodes.

Of the four classes of compounds detected in potato root exudates, the amino acids and phytohormones had the least effect on egg hatch. Although these compounds are also polar in nature, they are associated with the root exudates of several plants, including tomato (Kirwa et al., 2018); maize (Carvalhais et al., 2011), sorghum and cowpea (Odunfa, 1979), cotton (Sulochana, 1962), rice (Bacilio-Jiménez et al., 2003), and peanut (Li et al., 2013) among others. As such, they may be perceived by the PCN as non-specific chemo-stimulants. However, it is possible that they may contribute as important background signals to the SGAs and their aglycones in the root exudate to stimulate PCN hatching, which should be investigated. Further testing of the naturally occurring concentrations of the SGAs, aglycones, amino acids, phytohormones and unidentified compounds may shed more light on the role of the potato root exudate and its metabolites in PCN hatching.

## Conclusion

The results of the present study have significant implications for PCN management in East Africa. Since potato production in East Africa is dominated by small holder farmers, who find it difficult to adopt the available PCN management strategies, use of Solanaceae plants such as the indigenous edible African nightshade species *Solanum villosum* and *Solanum scabrum* can be promising trap crops for PCN and in the context of subsistence agriculture in SSA. In field trials, these two species were found to reduce PCN densities by up to 80% after three seasons (Chitambo et al., 2019). We recommend further studies on these trap crops to elucidate the underlying mechanisms as to how they work. A previous study has shown that the African nightshade species *Solanum sarrachoides* contains high levels of SGAs (Jared et al., 2015), which could potentially be used as a trap crop for PCN. Additionally, such plants can also be used as organic amendments, processed plant products or incorporated in a crop rotation system to reduce PCN levels in farmer crop fields (Torto et al., 2018b). Finally, synthetic standards of the SGAs and steroidal alkaloids could be used to stimulate suicidal hatch in PCN infested fields before farmers plant their potato crops.

In summary, the current study has shown that understanding chemo-ecological interactions of PCN with host plants provides opportunities for identifying semiochemicals that can be exploited for their management.

## Data Availability Statement

The raw data supporting the conclusions of this article will be made available by the authors, without undue reservation, to any qualified researcher.

## Author Contributions

BT and LC designed the experiments. JO performed the experiments. JO and LC wrote the first draft of the manuscript. DC, BT, AH, MN, LC, and JO edited the manuscript and wrote the final manuscript. BT, LC, and DC contributed with the reagents and laboratory space where the experiments were carried out.

## Conflict of Interest

The authors declare that the research was conducted in the absence of any commercial or financial relationships that could be construed as a potential conflict of interest.
